# 

**Published:** 1921-09-10

**Authors:** 


### Pensions for War Disablement.

FINAL DATE OF APPLICATION.

The Ministry of Pensions calls attention to the fact that any new claim by officers, nurses, or men, and by widows and dependants to pension, grant, gratuity, or allowance in respect of disablement must be made within seven years after the termination of active service, or within seven years after the official date of the termination of the war (August 31, 1921), whichever date is the earlier. No new claims will be considered after the expiry of the prescribed period. Any claimant who desires to appeal to a Pensions Appeal Tribunal against the rejection of his (or her) claim to pension must do so within a period of twelve months after the date of the notification of the rejection of his (or her) claim, or after the date of the commencement of the Act (August 19, 1921), whichever is the later date.

### Health Week, October 1921.

This movement, under the auspices of the Royal Sanitary Institute, 90 Buckingham Palace Road, S.W., should appeal to a wide circle. Established in 1912, the object of Health Week is to focus public attention for one week in the year on matters of health, and to arouse that sense of personal responsibility for health without which all public work, whether by the Government or local authorities, must fall far short of its aims. It is proposed that the dominant idea for 1921 should be "Health, Happiness, and Efficiency," and the consideration of what each individual can do for himself and his neighbour in securing a healthy life. The Week will be held from October 9 to 15, and it is hoped to secure the co-operation on local committees of the medical profession, nurses, teachers, and the various local authorities. In 1920 Health Week was celebrated in at least 100 centres and observed in 250 others.

### New Methods for Infectious Diseases.

In making a suggestion for more accommodation for the treatment of cases of measles, Dr. Macmillan, the Woolwich medical officer of health, considers it a debatable point whether or not the best use is being made of the existing infectious diseases beds, which are in the main reserved for cases of diphtheria and scarlet fever. He holds that many cases of scarlet fever can be adequately and safely treated at home, and he would like to see more of the existing accommodation in Metropolitan Asylums Board hospitals made available for the treatment of measles cases at the expense of reducing the number of beds maintained for the isolation of scarlet fever. This provision would materially lessen the death-rate and the complication rate of this disease.

### Medical Women in India.

To encourage women students in India to adopt the medical profession, the Government of Bombay has instituted four scholarships, tenable for one year at an Arts College, and then for a five-year full course at the Grant Medical College, Bombay. A scholar from Sind will have an option of joining the Lady Hardinge Medical College, Delhi.

### Trained Nurses for Child-Welfare Schemes.

Southwark Borough Council is endeavouring to induce the Minister of Health to revoke his decision not to sanction the appointment by the Borough Council of extra nurses and a mothers' help for maternity and child-welfare work. The Ministry maintains that the appointment of two nurses for the nursing of sick children attending the centres does not come within the scope of "nursing" as defined by the Local Government Board's circulars M and C W 4, dated August 9, 1918. Dr. G. Millson, the Medical Officer of Health, on the other hand, deplores this attitude. In a report to the Council he urges as an example the value of early treatment of infants suffering from specific disease, and the detection of commencing or early rickets. It is little use detecting specific disease and the early symptoms of rickets unless the proper treatment and nursing are carried out. This can only be done by the nurse. It is impossible, without neglecting her special work, for a health visitor, whose energies are sufficiently taxed by her ordinary duties, to carry out this nursing. In Dr. Millson's opinion it is nothing short of a calamity to build up a huge machine like the maternity and child-welfare scheme, at a great expense, without completing it by the inclusion of nursing. Dr. Millson's report has been forwarded to the Minister of Health.

### Schedule of Maternity Home Fees.

The Maternity Committee of the Fulham Borough Council has compiled a schedule of charges at the Maternity Home to meet the cases of patients desiring admission on the ground of insufficient accommodation in their own homes owing to the housing shortage. Many of these people are in a position to pay a higher fee than the £1 per week maternity benefit, and the scale subjoined is for such cases:

Husband and wife (income of £2 and under).—£1 a week.

Ditto (exceeding £2).—£1, and 50 per cent. of weekly income above £2, to a maximum of £4.

Husband and wife and one child (£2 10s. and under).—£1.

Ditto (exceeding £2 10s.).—£1, and 50 per cent. of weekly income above £2 10s.

Husband and wife and two children (£3).—£1.

Ditto (exceeding £3).—£1, and 50 per cent. of weekly income above £3.

Husband and wife and three children (£3 8s.).—£1.

Ditto (exceeding £3 8s.).—£1, and 50 per cent. of weekly income above £3 8s.

Husband and wife and four children (£3 16s.).—£1.

Ditto (exceeding £3 16s.).—£1, and 50 per cent. of weekly income above £3 16s.

Husband and wife and five children (£4 4s.).—£1.

Ditto (exceeding £4 4s.).—£1, and 50 per cent. of weekly income above £4 4s.

### Opening of Grosvenor Telephone Exchange.

A new public telephone exchange named Grosvenor was opened on September 3, at 2 p.m. The address of the new exchange is 39 South Audley Street, Oxford Street, W. 1, and, as it is within the five-mile circle from Oxford Circus, it is within the local fee area of all exchanges within a radius of ten miles from Oxford Circus. Grosvenor Exchange, which has up-to-date central battery equipment, will start with the transfer of 850 and 110 lines from Mayfair and Gerrard Exchanges respectively, and two weeks later another batch of 200 lines will be transferred from Mayfair. Whilst it is desirable that callers for the transferred subscribers should ask for the new Grosvenor numbers as soon as they are known to them, arrangements have been made to transfer calls made for the old numbers with as little trouble and delay as possible, the callers being advised of the change in each case.

### Passing Events and Topics.

### The Alterations at Shrewsbury.

Some welcome alterations and additions have been made at the Eye, Ear, and Throat Hospital, Shrewsbury. By the purchase of land, the rearrangement of the basement, and the extension of some upper rooms, the accommodation has been increased to admit fifty instead of thirty-two in-patients. The kitchen has been moved from the top landing to the basement, where, we understand, a laboratory and a mortuary and maids' sitting-room are now situated. The Chairman of the Committee, Mr. H. F. Herries, suggested the scheme, which includes the purchase of two houses and gardens adjoining the hospital. One of these houses becomes the Nurses' Home.

### Sweetmeats for Children's Ward.

The late Mr. John Dyer, who passed away recently, was a most generous supporter of the Swansea Hospital and other charitable institutions, to which during his lifetime he devoted three-quarters of his income. A great lover of children, he believed in giving gifts to children which they could understand, and it was announced at the meeting of the Swansea Hospital Board last week that, following out this policy, he had entrusted money to realise 7s. 6d. to purchase sweets every Saturday for children who happened to be occupants of the Dyer Ward.

### A Sixty Years' Record.

Colonel Charles Brook, who was re-elected Vice-President of the Lincoln County Hospital at a recent meeting, can show a wonderful record of long connection with that hospital dating back to 1856, when he was a pupil at the age of eighteen. He was appointed surgeon to the hospital in 1864, and remained an active surgeon for thirty-six years. He was responsible in 1876 for the resolution calling for a new hospital, the foundation-stone of which was laid two years later. On Colonel Brook's retirement from the surgeoncy of the hospital his nephew was appointed, and carries on with Colonel Brook the family's connection.

### Dental Hygienists.

Twelve States in the United States of America have legalised the practice of women dental hygienists, whose work is limited to cleaning and polishing the teeth above the gums.

### A Maximum of Seventy Beds per Doctor.

How many hospital beds can one doctor supervise? That is the question raised in connection with the Willesden Municipal Hospital at the last meeting of the Health Committee of the Urban District Council. Dr. Stewart, the medical superintendent, attended, and made a statement as to the excessive work he was called upon to undertake, and asked for the appointment of another resident medical officer, making three doctors in all at the hospital. The Health Committee reports that the hospital estimates for the current financial year provide for a daily average of 136 patients throughout the year, but owing to the exceptional prevalence of scarlet fever the number is exceeded at the present time, and a recent report registered 193 patients in hospital, *plus* 15 babies. It recognises that with a greater number of patients than seventy a doctor is working under pressure, and in the circumstances it proposes the appointment, temporarily, of a *locum tenens* assistant medical officer at eight guineas a week, with board and lodging, so long as the number of patients, excluding babies, exceeds 140, or an average of seventy patients for each of the two doctors at present permanently employed.

The League of the Roses is organising a Flag Day in Islington in aid of the Great Northern Hospital, on Tuesday, 20th inst.

September 10, 1921] [Price Twopence

### The Hospital

**RATES OF SUBSCRIPTION** (post free, payable in advance).
**Three months, 3/3; six months, 6/6; twelve months, 13/-. (Should the cost of printing and paper as well as increased postage, necessitate alteration of these rates, the period paid for will be reduced proportionately on unexpired subscriptions.) THE HOSPITAL "Index" to Volume LXIX., October 1920 to March 1921, is now ready, price 1/1 per copy, post free.** Address The Manager, THE HOSPITAL, "The Hospital" Building, 28/29 Southampton Street, Strand, London, W.C. 2.

